# First record of *Wroughtonia* Cameron (Hymenoptera, Braconidae, Helconinae) from Borneo, with description of three new species

**DOI:** 10.3897/zookeys.1287.203269

**Published:** 2026-07-31

**Authors:** Cheng-jin Yan, Zhenhua Zu, Yu Ning, Andrew Polaszek

**Affiliations:** 1 College of Agriculture and Biotechnology, Wenzhou Vocational College of Science and Technology, Wenzhou 325006, China College of Agriculture and Biotechnology, Wenzhou Vocational College of Science and Technology Wenzhou China; 2 Science: Research, Natural History Museum, London, UK Science: Research, Natural History Museum London United Kingdom; 3 College of Advanced Agricultural Sciences, Zhejiang A&F University, Hangzhou 311300, China College of Advanced Agricultural Sciences, Zhejiang A&F University Hangzhou China

**Keywords:** Braconid wasps, identification key, new record, new taxa, Oriental region, parasitoids, systematics

## Abstract

The wood-boring endoparasitoid beetle genus *Wroughtonia* Cameron (Hymenoptera: Braconidae: Helconinae) is quite diverse in the Oriental region, yet its equatorial fauna remains poorly understood. In this study, the genus is reported for the first time from Borneo. Three new species from Brunei are described and illustrated: *Wroughtonia vermiculata* Yan & Polaszek, **sp. nov**., *W. belalongensis* Yan & Polaszek, **sp. nov**. and *W. variivena* Yan & Polaszek, **sp. nov**. A key to the species of *Wroughtonia* from Borneo is provided.

## Introduction

*Wroughtonia* (Hymenoptera, Braconidae, Helconinae), established by Cameron in 1899, is a medium-sized genus including 58 valid species distributed in the Palaearctic, Nearctic and Oriental regions, with its highest regional diversity recently documented in China and Vietnam (Yu et al. 2016; Yan et al. 2017; Long et al. 2020; Hirose et al. 2023; Oanh et al. 2024). However, the equatorial parts of its Southeast Asia range remain a major taxonomic gap. As far as known *Wroughtonia* species are koinobiont endoparasitoids of Coleoptera larvae in wood, especially of Cerambycidae and Buprestidae (Viereck 1912; Gupta and Sharma 1976; Marsh 1979; van Achterberg 1987; Sheng 1990; Chou and Hsu 1998; Long et al. 2020; Oanh et al. 2024); however, the biology of all Bornean species remains unknown.

During our study of Helconinae, species of the genus *Wroughtonia* from Borneo were discovered for the first time. Consequently, three new species, *Wroughtonia vermiculata* Yan & Polaszek, sp. nov., *W. belalongensis* Yan & Polaszek, sp. nov. and *W. variivena* Yan & Polaszek, sp. nov., were discovered, and a key to the species of this genus from Borneo is provided.

## Material and methods

The higher classification at the subfamily level follows Chen and van Achterberg (2019) and Sharanowski et al. (2011). The terminology and measurements used follow van Achterberg (1988, 1993). An additional source for the description of sculpture and setation is from Belokobylskij (1998). The following abbreviations are used: POL = postocellar line; OOL = ocular-ocellar line; OD = maximum diameter of lateral ocellus.

Descriptions and measurements were made using a Nikon SMZ800 stereomicroscope. All figures were taken and processed using a digital Zeiss AxioZoom camera combined with Helicon Focus software. The images were further processed with Adobe Photoshop® 2026.

The type specimens are deposited in the Natural History Museum, London, UK (NHMUK).

## Systematics

### Wroughtonia

Taxon classificationAnimaliaHymenopteraBraconidae

Cameron, 1899

E6EEDABF-A0C7-5A6E-9078-6B295D5EB3DF

Wroughtonia Cameron, 1899: 56; Watanabe 1972: 3; Gupta and Sharma 1976: 353; van Achterberg 1987: 276–278; Chou and Hsu 1998: 298–301; Belokobylskij 1998: 412–413, 418–419; Yan et al. 2017: 414 (redefinition of genus); Long et al. 2020: 4; Hirose et al. 2023: 30. Type species: Wroughtonia cornuta Cameron, 1899, by monotypy.
Duportia Kieffer, 1921: 129–140. Synonymized by Watanabe (1972). Type species: Duportia cincticornis Kieffer, 1921 [= Wroughtonia unicornis (Turner, 1918)], by monotypy.
Spasskia Belokobylskij, 1989: 26–27, 1998: 420; Singh et al. 2005: 95–96; Yan et al. 2014: 2. Type species: Spasskia sigalphoides Belokobylskij, 1989, by original designation. Synonymized by Yan et al. (2017).

#### Key to the species of *Wroughtonia* Cameron from Borneo

**Table:** 

1	Ovipositor sheath 1.9× longer than hind tibia; first metasomal tergite coarsely vermiculate-rugose (Fig. 1I); fore wing vein r-m shorter than vein 2-SR (Fig. 1G); hind tibia ivory in basal half and darkened in apical half (Fig. 1H)	***W. belalongensis* sp. nov**.
–	Ovipositor sheath 1.5–1.6× longer than hind tibia; first tergite less coarsely vermiculate- or punctate rugose (Figs 2J, 3I); fore wing vein r-m subequal to or longer than vein 2-SR (Figs 2G, 3G); hind tibia predominantly yellow	**2**
2	Mesoscutal lobes densely punctate (Fig. 2F); precoxal sulcus wide and deep, coarsely crenulate (Fig. 2E); propodeum with a large, well-defined areola and distinct transverse carina (Fig. 2J); fore wing pterostigma about 3.0× as long as wide	***W. variivena* sp. nov**.
–	Mesoscutal lobes sparsely punctate (Fig. 3F); precoxal sulcus narrow and shallow, with sparse punctures (Fig. 3E); propodeum without a large defined areola, posteriorly coarsely foveolate-rugose (Fig. 3F); fore wing pterostigma about 3.7× as long as wide	***W. vermiculata* sp. nov**.

### Wroughtonia belalongensis

Taxon classificationAnimaliaHymenopteraBraconidae

Yan & Polaszek sp. nov.

528D9FA6-543D-5F82-A8B0-05D3000A3859

https://zoobank.org/28101B2A-FA0F-4709-83FC-9CCEE2E22270

Fig. 1

#### Diagnosis.

Body length 7.3 mm, black; eye 2.5× length of temple in dorsal view; malar suture weakly impressed; POL:OD:OOL = 10:7:21; vertex and temple smooth and shiny; frons crest-shaped, elevated and smooth latero-dorsally, crenulate latero-ventrally, smooth dorso-medially and shiny, medio-anteriorly rugose with a protruding lamella, frontal depression deep anteriorly, and rather so posteriorly; face coarsely vermiculate-rugose; clypeus weakly convex, densely reticulate-punctate dorsally, ventrally depressed and nearly smooth, apico-ventrally truncate; notauli narrow and shallow, crenulate, posteriorly rugose-reticulate and with a medial carina; precoxal sulcus wide and deep, coarsely crenulate; metanotum with a complete median carina, posteriorly triangularly protruding; first tergite apically 1.5× wider than basally, reticulate-punctate, its dorsal carinae distinct medially; length of first tergite 1.4× its apical width.

**Figure 1.**
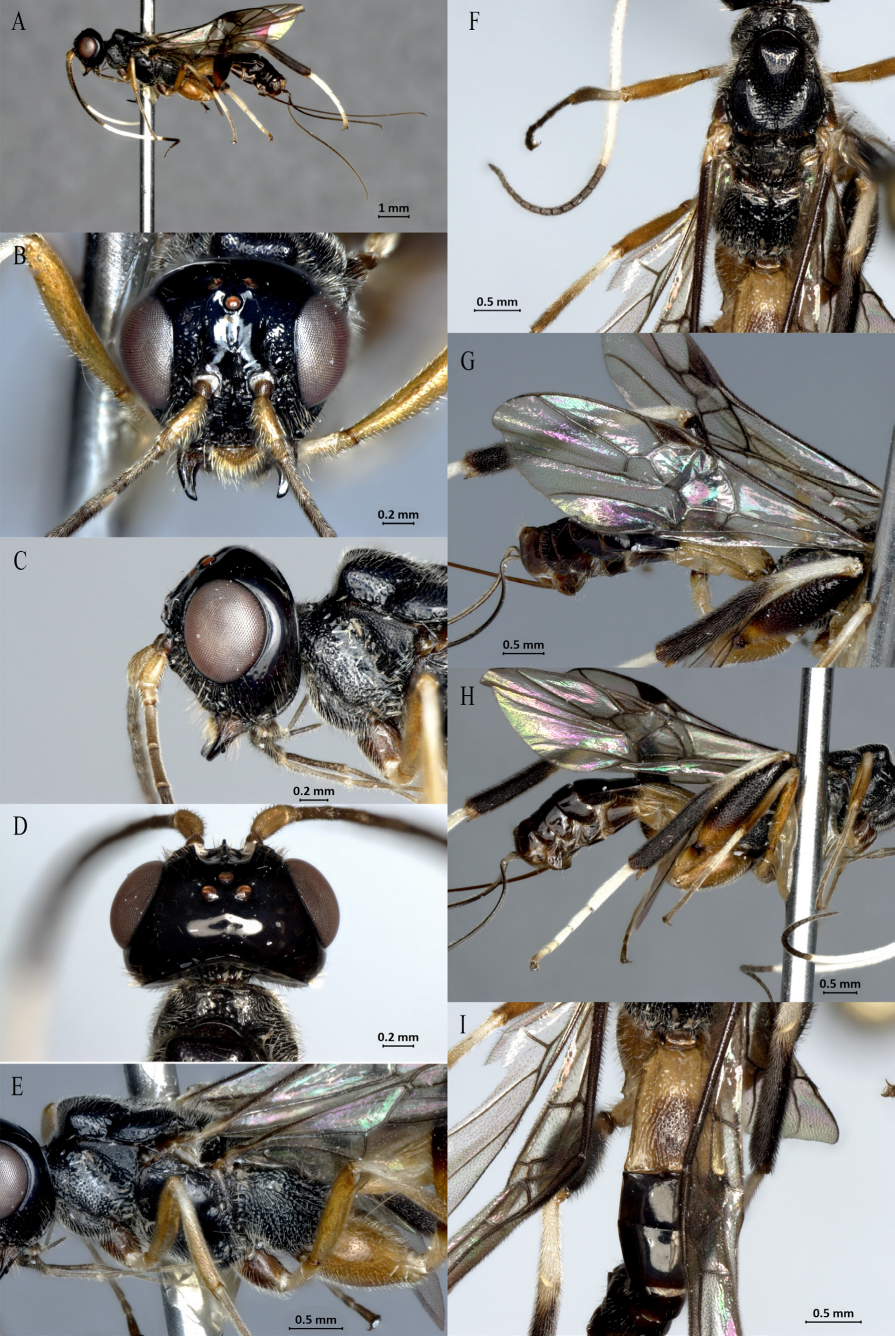
*Wroughtonia belalongensis* Yan & Polaszek, sp. nov. **A**. Habitus, lateral aspect; **B**. Head, anterior aspect; **C**. Head, lateral aspect; **D**. Head, dorsal aspect; **E**. Mesosoma, lateral aspect; **F**. Mesosoma, dorsal aspect; **G**. Fore wing; **H**. Hind leg, lateral aspect; **I**. Metasoma, dorsal aspect.

#### Description.

***Female***. Length of body (excluding ovipositor sheath) 7.3 mm, of setose part of ovipositor sheath 5.6 mm, of fore wing 5.6 mm (Fig. 1A).

**Head**. Antennomeres 35, third antennomere as long as fourth antennomere; length of third, fourth and penultimate antennomeres 4.3, 4.3 and 2.0× their width, respectively; length of maxillary palp 1.5× height of head; head 0.9× as long as wide in anterior view; length of eye 2.5× length of temple in dorsal view (Fig. 1D); length of malar space 1.1× basal width of mandible and 0.4× maximum width of eye; malar suture weakly impressed; POL:OD:OOL = 10:7:21; vertex and temple smooth and shiny; occipital carina complete, gradually curved ventrally and meeting hypostomal carina near base of mandible (Fig. 1C); frons crest-shaped, elevated and smooth latero-dorsally, crenulate latero-ventrally, smooth dorso-medially and shiny, medio-anteriorly rugose with a protruding lamella, frontal depression deep anteriorly, and rather so posteriorly; face (Fig. 1B) coarsely vermiculate-rugose; clypeus weakly convex, densely reticulate-punctate dorsally, ventrally depressed and nearly smooth, apico-ventrally truncate.

**Mesosoma**. Length of mesosoma 2.0× its height; pronope transverse and deep; sides of pronotum coarsely crenulate medially, remainder finely punctate; mesoscutal lobes densely punctate (Fig. 1E); notauli narrow and shallow, crenulate, posteriorly rugose-reticulate and with a medial carina (Fig. 1F); scutellar sulcus wide, with one carina and several lateral crenulae; scutellum densely punctulate, convex and without lateral carinae; precoxal sulcus wide and deep, coarsely crenulate; metanotum with a complete median carina, posteriorly triangularly protruding; propodeum narrowly smooth basally except some punctures, remainder mainly coarsely foveolate-rugose, with distinct transverse carina and short median carina.

**Wings. *Fore wing*** (Fig. 1G): 3.8× as long as wide. 1-M evenly curved; pterostigma 3.2× as long as wide; r:3-SR:SR1 = 10:12:67; 2-SR:3-SR:r-m = 15:10:10; 1-M:m-cu = 25:11; SR1 nearly straight; cu-a strongly inclivous and postfurcal; 1-CU1:2-CU1 = 7:30; r-m distinctly inclivous. ***Hind wing***: 1-M:1r-m = 14:20; cu-a nearly straight; marginal cell distinctly widened.

**Legs**. Length of fore tarsus 1.4× fore tibia; length of femur, tibia and basitarsus of hind leg 3.8 (without protuberance), 10.0 and 5.2× their width, respectively; outer and inner hind tibial spur 0.30 and 0.21× as long as basitarsus, respectively and outer spur widened; hind femur (Fig. 1H) moderately inflated and ventrally with transverse ridges; hind coxa stout.

**Metasoma**. First tergite (Fig. 1I) gradually widened posteriorly, apically 1.5× wider than basally, reticulate-punctate, its dorsal carinae distinct medially; length of first tergite 1.4× its apical width; second and following tergites smooth; second suture narrow and smooth; ovipositor sheath 1.7× as long as metasoma, 1.9× as long as hind tibia and 1.0× as long as fore wing.

**Colour**. Black; antenna dark brown, but 1^st^–2^nd^ antennomeres yellow-brown and 11^th^–20^th^ antennomeres ivory; palpi (including basal segments), tegula, pterostigma and most veins dark brown; fore and middle legs (except coxae red-brown), hind coxa and hind trochantellus yellow; hind femur (except narrowly yellow basally), hind tibia in apical half, second and following tergites red-brown; hind tibia in basal half and hind tarsus ivory; first tergite yellow except red-brown apico-medially; wing membrane faintly infumate; ovipositor sheath and setae dark brown with paler setae apically.

**Male**. Unknown.

#### Material examined (NHMUK).

***Holotype***: • ♀, Borneo, Brunei (4°34'N, 115°7'E), Kuala Belalong Field Studies Centre (Malaise [trap]), N Mawdsley coll., V. 1991. BMNH(E) 1991–173. No. NHMUK012850795.

#### Remarks.

This new species is similar to *W. sonla* Long from Vietnam but differs by having vein r-m of the fore wing shorter than vein 2-SR (vein r-m distinctly longer than vein 2-SR in *W. sonla*); the length of first tergite 1.4 times its apical width (first tergite about as long as its apical width in *W. sonla*); and the second tergite smooth (second tergite punctate-reticulate in *W. sonla*).

#### Host.

Unknown.

#### Etymology.

Named after the type locality of the holotype, Kuala Belalong Field Studies Centre, Brunei, Borneo.

#### Distribution.

Oriental (Borneo [Brunei]).

### Wroughtonia variivena

Taxon classificationAnimaliaHymenopteraBraconidae

Yan & Polaszek sp. nov.

119BC2F5-8E41-5ACA-A547-58848B8E851C

https://zoobank.org/E2C3830A-1019-4386-A687-3380EA35FD11

Fig. 2

#### Diagnosis.

Body length 9.0 mm, black; eye 2.3× length of temple in dorsal view; POL:OD:OOL = 10:9:18; vertex and temple smooth and shiny; frons crest-shaped, elevated, medially with a protruding lamella, smooth and shiny; face coarsely reticulate-punctate; clypeus densely reticulate-punctate dorsally, ventrally depressed and mainly smooth, apico-ventrally truncate; mesoscutal lobes densely punctate; notauli narrow and deep, crenulate, posteriorly rugose-reticulate and with a medial carina; propodeum smooth basally, behind with distinct transverse carina, mediobasal carina and large areola, areola smooth baso-medially, remainder and lateral areas of propodeum with transverse rugosity; first tergite apically 1.4× wider than basally, reticulate-punctate mostly except smooth apico-laterally and its dorsal carinae distinct in basal 0.7; length of first tergite 1.2× its apical width.

**Figure 2.**
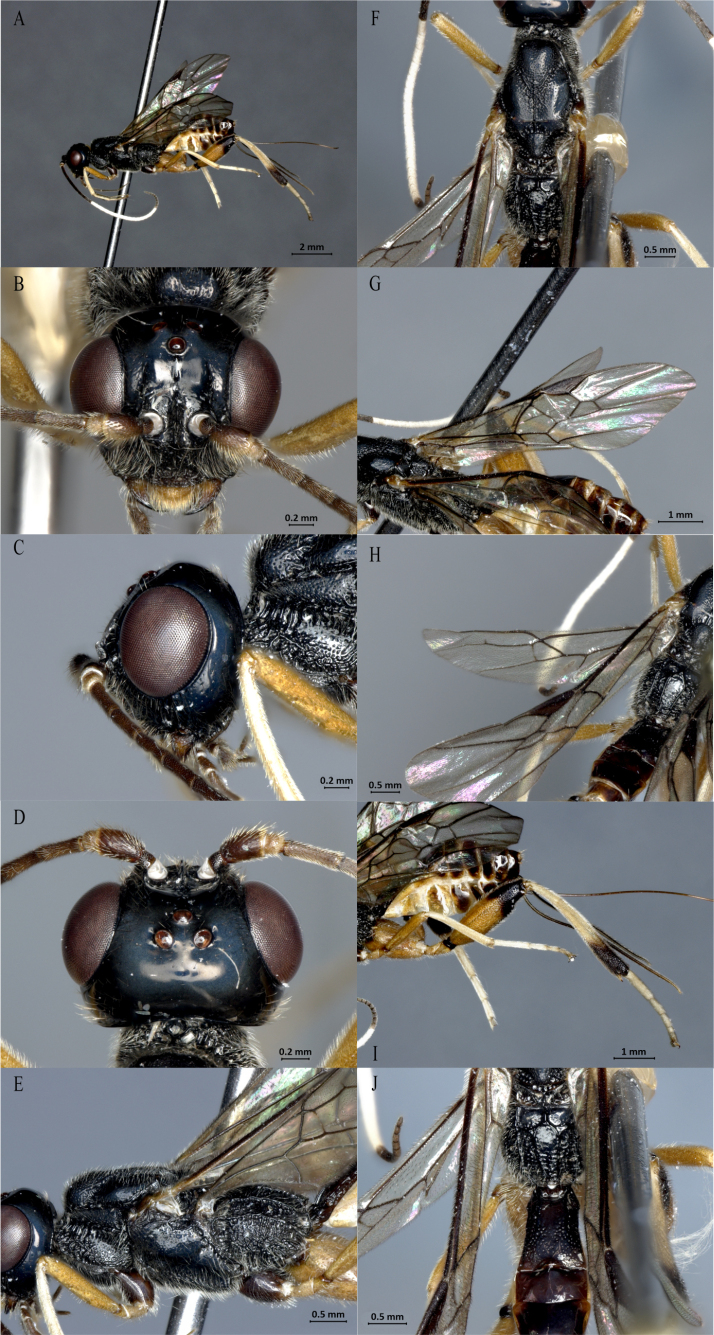
*Wroughtonia variivena* Yan & Polaszek, sp. nov. **A**. Habitus, lateral aspect; **B**. Head, anterior aspect; **C**. Head, lateral aspect; **D**. Head, dorsal aspect; **E**. Mesosoma, lateral aspect; **F**. Mesosoma, dorsal aspect; **G**. Fore wing; **H**. Hind wing; **I**. Hind leg, lateral aspect; **J**. Metasoma, dorsal aspect.

#### Description.

***Female***. Length of body (excluding ovipositor sheath) 9.0 mm, of setose part of ovipositor sheath 5.3 mm, of fore wing 6.8 mm (Fig. 2A).

**Head**. Antennomeres 35, third antennomere 1.1× as long as fourth antennomere; length of third, fourth and penultimate antennomeres 3.5, 3.3 and 1.4× their width, respectively; length of maxillary palp 1.7× height of head; head 0.8× as long as wide in anterior view; length of eye 2.3× length of temple in dorsal view (Fig. 2D); length of malar space 1.0× basal width of mandible and 0.5× maximum width of eye; malar suture moderately impressed; POL:OD:OOL = 10:9:18; vertex and temple smooth and shiny; occipital carina complete, gradually curved ventrally and meeting hypostomal carina far above base of mandible (Fig. 2C); frons crest-shaped, elevated, medially with a protruding lamella, smooth and shiny; face coarsely reticulate-punctate; clypeus densely reticulate-punctate dorsally, ventrally depressed and mainly smooth, apico-ventrally truncate (Fig. 2B).

**Mesosoma**. Length of mesosoma 2.1× its height; pronope round and deep; side of pronotum coarsely crenulate medially, reticulate-punctate ventrally, remainder finely punctate (Fig. 2E); mesoscutal lobes densely punctate; notauli narrow and deep, crenulate, posteriorly rugose-reticulate and with a medial carina; scutellar sulcus wide and with one medial and several lateral crenulae; scutellum densely punctulate, weakly convex and without lateral carinae; precoxal sulcus wide and deep, coarsely crenulate; metanotum with a complete median carina, posteriorly triangularly protruding; propodeum smooth basally, behind with distinct transverse carina, mediobasal carina and large areola, areola smooth baso-medially, remainder and lateral areas of propodeum with transverse rugosity (Fig. 2F).

**Wings**. ***Fore wing*** (Fig. 2G): about 4× as long as wide. 1-M evenly curved; pterostigma about 3.0× as long as wide; r:3-SR:SR1 = 7:6:43; 2-SR:3-SR:r-m = 12:6:9; 1-M:m-cu = 20:10; SR1 nearly straight; cu-a strongly inclivous and postfurcal; 1-CU1:2-CU1 = 5:30; r-m distinctly inclivous. ***Hind wing*** (Fig. 2H): 1-M:1r-m = 12:15; cu-a evenly curved; marginal cell distinctly widened. Venation asymmetrical, with one wing showing an additional closed cell.

**Legs**. Length of fore tarsus 1.5× fore tibia; length of femur, tibia and basitarsus of hind leg 3.3 (without protuberance), 7.9 and 5.5× their width, respectively; outer and inner hind tibial spurs 0.26 and 0.29× as long as basitarsus, respectively, and outer spur widened; hind femur moderately inflated and ventrally with transverse ridges (Fig. 2I); hind coxa stout.

**Metasoma**. First tergite gradually widened posteriorly, apically 1.4× wider than basally, reticulate-punctate mostly except smooth apico-laterally and its dorsal carinae distinct in basal 0.7 (Fig. 2J); length of first tergite 1.2× its apical width; second and following tergites smooth; second suture narrow and smooth; ovipositor sheath 1.5× as long as metasoma, 1.5× as long as hind tibia and 0.8× as long as fore wing.

**Colour**. Black; palpi (including basal segments), antenna (except 10^th^–25^th^ antennomeres ivory), fore and middle coxae, hind femur in apical 1/3 and hind tibia in apical 1/3 and tergites of metasoma red-brown; fore and middle femurs, fore and middle trochantelli, hind coxa, and remainder of hind femur yellow; fore and middle tibias, fore and middle tarsi, and remainder of hind tibia ivory; tegula, pterostigma and most veins dark brown; wing membrane faintly infumate; ovipositor sheath and setae dark brown with paler setae apically.

**Variation**. Body length 7.0–9.0 mm; 10^th^–23^rd^ antennomeres ivory. The holotype with a noticeable asymmetry between the left and right hindwing. However, this asymmetry is completely absent in all four paratypes, so this deviation is confirmed as an individual developmental aberration rather than a diagnostic character.

***Male***. Body length 6.3–7.2 mm; wing length 5.1–5.8 mm; antenna with 35–40 antennomeres, dark brown and with ivory band submedially (12^th^–21^st^ or 12^th^–23^rd^ antennomeres).

#### Material examined (NHMUK).

***Holotype***: • ♀, Borneo, Brunei (4°34'N, 115°7'E), Kuala Belalong Field Studies Centre (Malaise [trap]), N Mawdsley coll., V. 1991. BMNH(E) 1991–173. No. NHMUK012850796. ***Paratypes***: • 2♀♀ 2♂♂, same data as holotype, No. NHMUK012850797, 012850798, 012850799, 012850800.

#### Remarks.

This new species is similar to *Wroughtonia undulata* Long from Vietnam, but differs by having the dorsal carinae of the first tergite distinct in the basal 0.7 (dorsal carinae almost complete in *W. undulata*); the first tergite reticulate-punctate mostly except smooth apico-laterally (almost smooth or coriaceous in *W. undulata*); and the hind femur without a tooth-shaped protuberance (with ventral serrations and stout tooth-shaped protuberance in *W. undulata*).

#### Host.

Unknown.

#### Etymology.

The name is derived from “*varius*” (Latin for “variable”) and “*vena*” (Latin for “vein”), referring to the variable venation of the hind wing observed in the holotype.

#### Distribution.

Oriental (Borneo [Brunei]).

### Wroughtonia vermiculata

Taxon classificationAnimaliaHymenopteraBraconidae

Yan & Polaszek sp. nov.

A50BF6C9-3977-5DFD-AB3C-95A90F02CF71

https://zoobank.org/99E14DC9-C659-454E-AFE0-26BF5F5FC6FE

Fig. 3

#### Diagnosis.

Body length 12.3 mm, black; eye 1.6× length of temple in dorsal view; POL:OD:OOL = 8:9:20; vertex and temple smooth and shiny; frons crest-shaped elevated, dorso-medially smooth and shiny, medio-anteriorly weakly punctate with a protruding lamella, frontal depression deep anteriorly and less so posteriorly; face coarsely vermiculate-rugose; clypeus densely reticulate-punctate dorsally, ventrally depressed and mainly smooth, apico-ventrally truncate; mesoscutal lobes sparsely punctate; notauli wide and deep, crenulate, coarsely rugose-reticulate; precoxal sulcus narrow and shallow, with some spaced punctures; propodeum narrowly smooth basally except with some punctures, distinct transverse behind, and curved carina mainly coarsely foveolate-rugose, with irregular areola and with short median carina; first tergite apically 1.3× wider than basally, coarsely vermiculate-rugose and its dorsal carinae distinct in basal half; length of first tergite 1.2× its apical width; second tergite rugose but apically and medially smooth.

**Figure 3.**
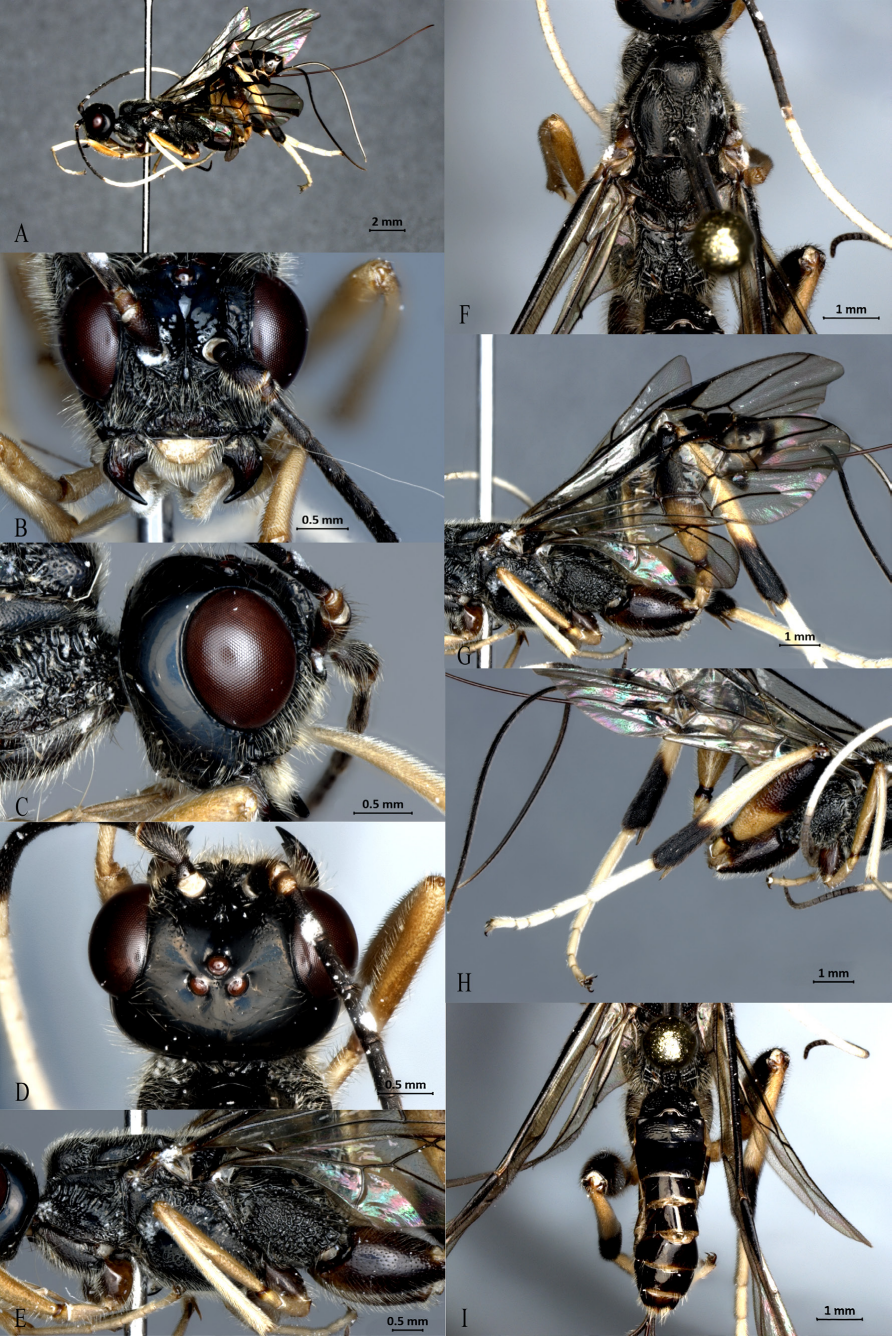
*Wroughtonia vermiculata* Yan & Polaszek, sp. nov. **A**. Habitus, lateral aspect; **B**. Head, anterior aspect; **C**. Head, lateral aspect; **D**. Head, dorsal aspect; **E**. Mesosoma, lateral aspect; **F**. Mesosoma, dorsal aspect; **G**. Wings; **H**. Hind leg, lateral aspect; **I**. Metasoma, dorsal aspect.

#### Description.

***Female***. Length of body (excluding ovipositor sheath) 12.3 mm, of setose part of ovipositor sheath 9.5 mm, of fore wing 10.3 mm (Fig. 3A).

**Head**. Antennomeres 39, third antennomere as long as fourth antennomere; length of third, fourth and penultimate antennomeres 3.3, 3.4 and 1.5× their width, respectively; length of maxillary palp 1.5× height of head; head 0.7× as long as wide in anterior view; length of eye 1.6× length of temple in dorsal view (Fig. 3D); length of malar space 0.7× basal width of mandible and 0.4× maximum width of eye; malar suture moderately impressed; POL:OD:OOL = 8:9:20; vertex and temple (Fig. 3C) smooth and shiny; occipital carina complete, gradually curved ventrally and meeting hypostomal carina far above base of mandible; frons crest-shaped elevated, dorso-medially smooth and shiny, medio-anteriorly weakly punctate with a protruding lamella (Fig. 3D), frontal depression deep anteriorly and less so posteriorly; face coarsely vermiculate-rugose; clypeus densely reticulate-punctate dorsally, ventrally depressed and mainly smooth, apico-ventrally truncate (Fig. 3B).

**Mesosoma**. Length of mesosoma 2.0× its height; pronope triangular; side of pronotum coarsely crenulate medially, reticulate-punctate ventrally, remainder finely punctate (Fig. 3E); mesoscutal lobes sparsely punctate; notauli wide and deep, crenulate, coarsely rugose-reticulate (Fig. 3F); scutellar sulcus wide and with one medial and several lateral crenulae; scutellum sparsely punctulate, nearly flat and without lateral carinae; precoxal sulcus narrow and shallow, with some spaced punctures; metanotum with a complete median carina, posteriorly triangularly protruding; propodeum narrowly smooth basally except with some punctures, distinct transverse behind, and curved carina mainly coarsely foveolate-rugose, with irregular areola and with short median carina.

**Wings. *Fore wing*** (Fig. 3G): about 4× as long as wide. 1-M evenly curved; pterostigma about 4.0× as long as wide; r:3-SR:SR1 = 7:10:39; 2-SR:3-SR:r-m = 10:10:9; 1-M:m-cu = 27:9; SR1 nearly straight; cu-a strongly inclivous and postfurcal; 1-CU1:2-CU1 = 2:15; r-m distinctly inclivous. ***Hind wing***: 1-M:1r-m = 8:14; cu-a slightly curved posteriorly; marginal cell distinctly widened.

**Legs**. Length of fore tarsus 1.5× fore tibia; length of femur, tibia and basitarsus of hind leg 3.7 (without protuberance), 8.0 and 4.0× their width, respectively; outer and inner hind tibial spurs 0.25 and 0.21× as long as basitarsus, respectively, and outer spur widened; hind femur moderately inflated and ventrally with transverse ridges (Fig. 3H); hind coxa stout.

**Metasoma**. First tergite gradually widened posteriorly, apically 1.3× wider than basally, coarsely vermiculate-rugose and its dorsal carinae distinct in basal half; length of first tergite 1.2× its apical width; second tergite rugose but apically and medially smooth (Fig. 3I); second suture narrow and smooth; third and following tergites smooth; ovipositor sheath 1.5× as long as metasoma, 1.6× as long as hind tibia and 0.9× as long as fore wing.

**Colour**. Black; palpi (including basal segments), fore and middle legs (except coxae red-brown), hind tibia (except narrowly black apically), hind trochantelli and hind tarsi yellow; hind femur red-brown in apical half and yellow in basal half; antenna dark brown, but 9^th^–22^nd^ antennomeres ivory; tegula, pterostigma and most veins dark brown; wing membrane faintly infumate; ovipositor sheath and setae dark brown with paler setae apically; metasoma yellow to dark brown ventrally and black dorsally.

**Variation**. Body length 10.7–12.3 mm; fore wing length 9.5–10.3 mm; antennomeres 37–39, 8^th^–21^th^ ivory.

***Male***. Unknown.

#### Material examined (NHMUK).

***Holotype***: • ♀, Borneo, Brunei (4°34'N, 115°7'E), Kuala Belalong Field Studies Centre (Malaise [trap]), N. Mawdsley coll., V. 1991. BMNH(E) 1991–173. No. NHMUK012850801. ***Paratype***: • 1♀, same data as holotype, No. NHMUK012850802.

#### Remarks.

This new species is similar to *W. vietnamica* Long from Vietnam, but differs by the dorsal carinae of the first tergite distinct in basal half (dorsal carinae in basal 0.7 of tergite in *W. vietnamica*); the first tergite coarsely vermiculate-rugose (first tergite smooth basally and apically, coriaceous medially, areolate-rugulose subapically and laterally in *W. vietnamica*); and the second tergite rugose but apically and medially smooth (second tergite smooth in *W. vietnamica*).

#### Host.

Unknown.

#### Etymology.

The name is derived from “*vermiculatus*” (Latin for “worm-like”) and refers to the strongly vermiculate-rugose sculpture of the face and the first metasomal tergite.

#### Distribution.

Oriental (Borneo [Brunei]).

## Discussion

The discovery of three new species from Brunei marks the first record of the genus *Wroughtonia* from Borneo. Historically, the documented diversity of the genus *Wroughtonia* in the Oriental region has been mostly concentrated in its northern and western continental areas (such as southern China, Vietnam, India and Myanmar). Prior to this study, the southern equatorial parts of the region remained almost entirely blank. The sympatric occurrence of three morphologically distinct species in a localized survey in Borneo suggests that the previously assumed low species richness in the southern Oriental region is more likely a result of under-sampling rather than a true biological pattern.

## Supplementary Material

XML Treatment for Wroughtonia

XML Treatment for Wroughtonia belalongensis

XML Treatment for Wroughtonia variivena

XML Treatment for Wroughtonia vermiculata

## Additional information

### Artificial Intelligence (AI) use

The authors accept full responsibility for the content of the manuscript, including the disclosure of any use of AI.

No AI tools were used in the preparation of this manuscript.

### Author ORCIDs

C.-j. Yan https://orcid.org/0000-0001-5723-9635

A. Polaszek https://orcid.org/0000-0002-7171-3353

### Data availability

All of the data that support the findings of this study are available in the main text.

## References

[B1] Belokobylskij SA (1989) New and little known taxa of braconids of the subfamily Helconinae (Hymenoptera, Braconidae) from the Far East of the USSR. Proceedings of the Zoological Institute 188: 23–38.

[B2] Belokobylskij SA (1998) Subfam. Helconinae, In: Lehr PA (Ed.) Opredelitel’ nasekomykh Dal’nego Vostoka Rossii. Tom 4. Setchatokryloobraznye, skorpionnitzy, pereponchatokrylye. Chast’ 3. Dal’nauka, Vladivostok, 441–468. [in Russian]

[B3] Cameron P (1899) Hymenoptera Orientalia, or contributions to a knowledge of the Hymenoptera of the Oriental Zoological Region. Part VIII. The Hymenoptera of the Khasia Hills. First paper. Memoirs and Proceedings of the Manchester Literary and Philosophical Society 43(3): 1–220.

[B4] Chen XX, van Achterberg C (2019) Systematics, phylogeny and evolution of braconid wasps: 30 years of progress. Annual Review of Entomology 64: 335–358. 10.1146/annurev-ento-011118-111856 30332295

[B5] Chou LY, Hsu TC (1998) The Braconidae (Hymenoptera) of Taiwan 8. Brulleiini, Diospilini and Helconini. Journal of Agricultural Research of China 47(3): 283–314.

[B6] Gupta VK, Sharma V (1976) Studies on *Wroughtonia* (Hymenoptera: Braconidae: Helconinae). Oriental Insects 10(3): 353–362. 10.1080/00305316.1976.10432334

[B7] Hirose Y, Fujie S, Maeto K (2023) New records of genus *Wroughtonia* (Hymenoptera: Braconidae: Helconinae) from Japan. Japanese Journal of Systematic Entomology 29(1): 29–36.

[B8] Kieffer JJ (1921) Sur divers hymenoptères destructeurs des cerambycides nuisibles au cafeier et au bambou. Bulletin Agricole de l’Institut de Saigon 3: 129–140.

[B9] Long KD, van Achterberg C, Carpenter JM, Oanh NT (2020) Review of the genus *Wroughtonia* Cameron, 1899 (Hymenoptera, Braconidae, Helconinae), with the description of 12 new species from Vietnam. American Museum Novitates 3953: 1–54. 10.1206/3953.1

[B10] Marsh PM (1979) Braconidae. Aphidiinae. Hybrizontidae. In: KV Crombein, PD Hurd, Jr DR Smith, BD Burks (Eds) Catalog of Hymenoptera in America north of Mexico. Smithsonian Institution Press, Washington DC, 144–313.

[B11] Oanh NT, Long KD, Pham NT, Mai PQ, Nghiep HT (2024) Addition to the review of the genus *Wroughtonia* Cameron, 1899 (Hymenoptera, Braconidae, Helconinae) from Vietnam: Description of two new species. Zootaxa 5446(2): 221–235. 10.11646/zootaxa.5446.2.4 39645880

[B12] Sharanowski BJ, Dowling APG, Sharkey MJ (2011) Molecular phylogenetics of Braconidae (Hymenoptera: Ichneumonoidea), based on multiple nuclear genes, and implications for classification. Systematic Entomology 36: 549–572. 10.1111/j.1365-3113.2011.00580.x

[B13] Sheng ML (1990) New records of Hymenoptera parasitizing wood-boring insects from China. Entomotaxonomia 12(1): 56.

[B14] Singh S, Belokobylskij SA, Chauhan N, Pande S (2005) Description of a new species of the genus *Spasskia* Belokobylskij, 1989 (Hymenoptera, Braconidae) from India, with first record of the genus in the Oriental Region. Annales Zoologici (Warszawa) 55(1): 95–98. 10.3161/0003454053642194

[B15] Turner RE (1918) Notes on the Braconidae in the British Museum. IV. On new Helconinae, mostly Australian. Annals and Magazine of Natural History (9) 2: 163–173. 10.1080/00222931808562359

[B16] van Achterberg C (1987) Revision of the European Helconini (Hymenoptera, Braconidae, Helconinae). Zoologische Mededelingen Leiden 61: 263–285.

[B17] van Achterberg C (1988) Revision of the subfamily Blacinae Foerster (Hymenoptera, Braconidae). Zoologische Verhandelingen (Leiden) 249: 1–324.

[B18] van Achterberg C (1993) Illustrated key to the subfamilies of the Braconidae (Hymenoptera: Ichneumonoidea). Zoologische Verhandelingen Leiden 283: 1–189.

[B19] Viereck HL (1912) Contributions to our knowledge of bees and ichneumon-flies, including descriptions of 21 new genera and 57 new species of ichneumon-flies. Proceedings of the United States National Museum 42: 613–648. 10.5479/si.00963801.42-1920.613

[B20] Watanabe C (1972) A revision of the Helconini of Japan and review of Helconinae genera of the world (Hymenoptera: Braconidae). Insecta Matsumurana 35: 1–18.

[B21] Yan CJ, He JH, Chen XX (2014) The discovery of the genus *Spasskia* Belokobylskij, 1989 (Hymenoptera: Braconidae) in China, with description of a new species. Journal of Insect Science 14(119): 1–5. 10.1673/031.014.119 PMC4222300 25368063

[B22] Yan CJ, van Achterberg C, He JH, Chen XX (2017) Review of the tribe Helconini Foerster s.s. from China, with the description of 18 new species. Zootaxa 4291(3): 401–457. 10.11646/zootaxa.4291.3.1

[B23] Yu DS, van Achterberg C, Horstmann K (2016) Taxapad 2016. World Ichneumonoidea 2016. Taxonomy, Biology, Morphology and Distribution. Vancouver, Canada.

